# RNAi of Neuropeptide CCHamide-1 and Its Receptor Indicates Role in Feeding Behavior in the Pea Aphid, *Acyrthosiphon pisum*

**DOI:** 10.3390/insects15120939

**Published:** 2024-11-28

**Authors:** Sohaib Shahid, Muhammad Bilal Amir, Tian-Bo Ding, Tong-Xian Liu, Guy Smagghe, Yan Shi

**Affiliations:** 1 Shandong Engineering Research Center for Environment-Friendly Agricultural Pest Management, College of Plant Health and Medicine, Qingdao Agricultural University, Qingdao 266109, China; sohaibshahid90@hotmail.com (S.S.); dr.mbilalamir@outlook.com (M.B.A.); tbding@qau.edu.cn (T.-B.D.); txliu@qau.edu.cn (T.-X.L.); 2 State Key Laboratory of Integrated Management of Pest Insects and Rodents, Institute of Zoology, Chinese Academy of Sciences, Beijing 100101, China; 3 University of Chinese Academy of Sciences, Beijing 100049, China; 4 Institute of Entomology, Guizhou University, Guiyang 550025, China; guysma9@gmail.com; 5 Cellular and Molecular Life Sciences, Department of Biology, Vrije Universiteit Brussel (VUB), 1050 Brussels, Belgium; 6 Department of Plants and Crops, Ghent University, 9000 Ghent, Belgium

**Keywords:** *Acyrthosiphon pisum*, neuropeptide, *CCHamide-1*, feeding behavior, parthenogenetic reproduction

## Abstract

Neuropeptide *CCHamide-1* (abbreviated as *CCHa1*) is a recently discovered peptide that is present in many arthropods and is the ligand of the *CCHa1R*, a member of the G protein-coupled receptors (GPCRs) superfamily, which plays a regulatory role in diverse physiological processes such as feeding, circadian rhythm, insulin production, lipid metabolism, growth, and reproduction. However, the function of this gene in aphids is still unknown. Here, we characterized and determined the potential role of *CCHa1*/*CCHa1R* signaling in the pea aphid, *Acyrthosiphon pisum*, which is a notorious pest in agriculture. The docking analysis revealed that the *CCHa1* peptide binds to its receptor *CCHa1R* through specific amino acid residues, which are critical for maintaining the structural and functional integrity of the peptide–receptor complex. Quantitative real-time reverse transcription-PCR (qRT-PCR) revealed the expression levels of *CCHa1*/*CCHa1R* transcripts in different development stages and different tissues, indicating that the *CCHa1* expression was high in the first nymphal instar compared to the upcoming nymphal instars and adults, and was predominantly high in the brain. The *CCHa1*/*CCHa1R* transcript levels were significantly upregulated in starved aphids compared to fed aphids. Moreover, RNAi knockdown by the injection of dsRNA-*CCHa1* and dsRNA-*CCHa1R* significantly reduced the corresponding expression of the target gene and reduced their food intake in adult aphids, as revealed by the electrical penetration graph results. *CCHa1*/*CCHa1R*-silencing also reduced the reproduction, but not the survival, in *A. pisum*. Our data demonstrated that *CCHa1/CCHa1R* play a role in the regulation of feeding in *A. pisum*, suggesting a role of the *CCHa1* signaling pathway in the aphids relating to their nutritional status.

## Simple Summary

G protein-coupled receptors (GPCRs) are responsible for the activity of neuropeptides, and interestingly, a number of GPCRs are well-known as pharmaceutical targets, and some are currently being considered as potential pest insect control targets. The purpose of this study is to investigate the function of *CCHamide-1* (abbreviated as *CCHa1*) and its receptor *CCHamide1-receptor* (abbreviated as *CCHa1R*), which belongs to the GPCR superfamily in pea aphid, *Acyrthosiphon pisum*. The pea aphid is a serious agricultural pest and serves as a biological model for various studies. The silencing of *CCHa1* significantly reduced the aphid feeding behavior and reproduction, but not its survival.

## 1. Introduction

Neuropeptides regulate physiological, developmental, and behavioral processes throughout the life cycle of animals by acting as neuroactive signaling molecules [1,2,3]. They exert their function by binding as ligands with ligand-binding pockets of cognate G protein-coupled receptors (GPCRs), which are the largest superfamily of cell surface membrane receptors and are encoded by approximately 1000 genes, sharing conserved seven-transmembrane (7TM) helices connected by three intra- and three extracellular loops [4,5,6,7]. This binding initiates the signaling transduction, which triggers a cascade of events within the cell, leading to the activation of G proteins and subsequent downstream signaling pathways. Subsequently, they modulate intercellular interaction and initiate biological effects that influence various physiological functions and behaviors. Several GPCRs are well-known as pharmaceutical targets, and some are currently being considered as potential pest insect control targets [8,9,10].

The *CCHamide* gene was initially identified within the genome of the silk worm, *Bombyx mori*, as a novel neuropeptide in 2008 [11]. These authors proposed the nomenclature “*CCHa*” for this newly identified neuropeptide, since this proposal is based on the observation that the peptide contains two conserved cysteine residues and a C-terminal amidated histidine residue (XCXXXXXXCXXXHamide), which are features observed in a majority of the analyzed species [11]. Subsequently, in 2010, two *CCHa* genes, which encode two precursors, namely *CCHa1* and *CCHamide-2* (*CCHa2*), were identified in the pea aphid, *Acyrthosiphon pisum* [12]. Moreover, in 2011 and 2012, initial deorphanization in the fruit fly, *Drosophila melanogaster*, of two GPCRs, encoded by CG14593 and CG30106, showed these as the first *CCHa* receptors, and this was reported together with the isolation of *CCHa1* and *CCHa2* precursors [13,14]. In insects, two *CCHa* receptors exist, *CCHa1R* and *CCHa2R*. In *D. melanogaster*, activation of the *CCHa* receptors is triggered by either *CCHa1* or *CCHa2* precursors, suggesting that these neuropeptide signaling systems have different functions [13].

The function of *CCHa2* and its receptor, *CCHa2-R*, has been elucidated in various insects, including the regulation of feeding behaviors observed in the black blowfly (*Phormia regina*), fruit fly (*D. melanogaster*), and pea aphid (*A. pisum*) [14,15,16,17]. For *CCHa1*, it has been reported to localize within the olfactory neurons of the CNS and the endocrine cells of the midgut in *D. melanogaster*. These endocrine cells have the appearance of sensory cells, projecting processes close to or into the gut lumen [17,18]. The silencing of *CCHa1* in Or59b-expressing olfactory neurons abolished the starvation effect in *D. melanogaster* [16]. Given its documented presence in the CNS and midgut of insect species, *CCHa1* has also been termed the brain–gut peptide, or enteroendocrine peptide [13,14,19,20,21,22,23]. Furthermore, *CCHa1* and *CCHa2* have been detected in the midgut and brain of male and female mosquitoes of *Aedes aegypti* [23]. The *dsCCHa2*-mediated knockdown revealed its role in regulating the feeding in female mosquitoes, as studied by a capillary feeder bioassay [23]. Despite the research on the *CCHa1* signaling system in *D. melanogaster* and *A. aegypti*, information regarding the functional roles and localization of *CCHa1* and its receptor, *CCHa1R*, in aphids is lacking.

Plant phloem-feeding insects like aphids are characterized by their soft bodies and hemimetabolous life cycle, and are notorious for causing significant damage to a wide range of horticultural crops [24]. They have a remarkable ability to reproduce rapidly and adapt to diverse environments, which is the primary reason behind their devastating impact, causing significant crop losses through both direct sap-feeding and the transmission of viral diseases [25,26]. *A. pisum* has emerged as a valuable model organism for a wide range of scientific investigations. The completion of its whole-genome sequence has facilitated the development of new molecular tools for aphid control [24]. The International Aphid Genomic Consortium has selected *A. pisum* as the model aphid species for developing genomic resources such as ESTs or genome sequencing [25,26]. Hence, elucidating the physiology and behavior modulated by neuropeptides in *A. pisum* presents promising avenues that can be extrapolated to other aphid species, facilitating a comprehensive understanding of mechanisms and the development of effective control measures.

Our research investigates the expression and functional role of *CCHa1*/*CCHa1R* in *A. pisum*. Firstly, the relative expression of *CCHa1*/*CCHa1R* in different developmental stages and tissues of *A. pisum* was assessed using qRT-PCR. Moreover, we tested what the effect was of starvation and feeding on the expression of *CCHa1*/*CCHa1R*. In addition to elucidate the potential role of *CCHa1* in regulating the feeding behavior in *A. pisum*, we employed RNA interference (RNAi) to silence the transcripts of *CCHa1*/*CCHa1R*, and subsequently analyzed feeding behavior using the electrical penetration graph (EPG) technique. The results revealed that silencing *CCHa1*/*CCHa1R* significantly impacted probing behavior and phloem feeding, ultimately inhibiting food consumption and lowering reproductive rates in *A. pisum*.

## 2. Materials and Methods

### 2.1. Insect Rearing

Wingless pea aphid populations were established on potted broad bean plants (*Vicia faba*) at the Laboratory of Insect Ecology and Molecular Biology, College of Plant Health and Medicine, Qingdao Agricultural University, Shandong, China, and the pea aphid colonies were propagated asexually at 21 ± 2 °C with 70 ± 5% relative humidity and exposed to 16 h of light followed by 8 h of darkness daily, as reported previously [17]. In brief, a low-density rearing strategy, with about 5 aphids per plant, was employed to prevent the development of a winged population and competition. To ensure synchronicity generation for experiments, nymphs aged 0–12 h were carefully collected and meticulously transferred onto fresh broad bean leaves.

### 2.2. Primers, Sequences, and Phylogenetic Analysis

We designed the optimal primers required for quantitative polymerase chain reaction (qPCR) assays using Primer 3 (https://bioinfo.ut.ee/primer3-0.4.0/ accessed on 19 December 2023), and these primers were subsequently synthesized by Sangon Biotech (Shanghai, China). The primer sequence used in this study is mentioned in Table S1. From the published literature on *A. pisum*, neuropeptide and neurohormone precursors [12], as well as GPCRs [27], cDNA sequences encoding *CCHa1*/*CCHa1R*, were obtained. The open reading frame (ORF) for the precursor and receptor were confirmed through the ORF finder (https://www.ncbi.nlm.nih.gov/orffinder/ accessed on 10 January 2024). For the precursor, the signal peptides were predicted using the SignalP-5.0server (http://www.cbs.dtu.dk/services/SignalP/ accessed on 22 January 2024), and the sequence logo of the C-terminal motif of *CCHa1* was made using Weblogo [28]. To visualize the evolutionary relationships between *CCHa1*/*CCHa1R* in *A. pisum* and other insects, we constructed a neighbor-joining tree with 1000 bootstrap replicates. The phylogenetic analysis was executed in MEGA 5.2, following the method outlined by [29,30]. The chosen model was P-distance, and pairwise deletion was employed for gaps and missing data treatment [29,30]. The accession number of the sequences used to visualize the evolutionary relationship of *CCHa1* with other insects is mentioned in Table S2, and *CCHa1R* is mentioned in Table S3. To predict the transmembrane domains of *CCHa1R*, we employed TMHMM version 2.0 (http://www.cbs.dtu.dk/services/TMHMM/ accessed on 15 February 2024), ensuring accuracy in our structural analysis.

### 2.3. Modeling CCHa1R Docked with Mature Peptide CCHa1

Despite utilizing the SWISS-MODEL platform for homology modeling, we were unable to identify suitable templates with amino acid sequence similarity exceeding 30%. In response to the initial challenges, we adopted an alternative strategy, employing a folded gender approach via the iTASSER platform. This methodology is particularly adept at addressing the challenges posed by proteins with minimal sequence homology and a dearth of appropriate templates. We employed the GPCR I-TASSER (Iterative Threading ASSEmbly Refinement) server to construct the structural model of the *CCHa1R*. Model reliability was evaluated using TM-score values, a structural similarity coefficient ranging from 0 to 1. Scores exceeding 0.5 indicate correct topological structure and reliability, while scores below 0.17 suggest a random, unreliable model. The homology model of the *CCHa1R*, with its C-terminal region excised, served as the basis for docking studies with the *CCHa1* mature peptide. We employed a two-step docking approach, first utilizing the Induced Fit Docking (IFD) method to generate preliminary results, followed by refinement using the QM-Polarized Ligand Docking method. We executed the docking simulations using the Maestro software platform. The previously established docking model of the *CCHa1* mature peptide and *CCHa1R* served as the foundation for our subsequent simulation studies. The docking model of the receptor-mature peptide complex was integrated into a lipid bilayer using the CHARMM-GUI platform. The system was balanced by the addition of chloride ions, ensuring overall charge neutrality. Molecular docking studies, focusing on the interaction between *CCHa1* and *CCHa1R* binding sites, were executed using PyMOL.

### 2.4. RNA Extraction and cDNA Synthesis

We isolated RNA using the TRIzol^®^ reagent (Invitrogen, Carlsbad, CA, USA), adhering strictly to the manufacturer’s recommended protocol. To ensure RNA quality and quantify concentrations accurately, we utilized a NanoDrop 2000 spectrophotometer (Thermo Fisher, Waltham, MA, USA) for quality checks. For the elimination of genomic DNA contamination, RNA from each sample was subjected to treatment with gDNA Eraser and 5X gDNA Eraser Buffer, a protocol meticulously followed according to the guidelines provided by Takara in Dalian, China. Subsequently, cDNA synthesis was achieved using the PrimeScript^TM^ RT Reagent Kit with gDNA Eraser from Takara (Dalian, China), following the manufacturer’s recommended procedure. To prepare the cDNA for quantitative Reverse Transcription Polymerase Chain Reaction (qRT-PCR), a 10-fold dilution procedure was implemented to ensure optimal concentrations for subsequent analyses.

### 2.5. Real-Time Quantitative PCR Analysis

We processed samples to quantify the mRNA expression levels of target genes *CCHa1*/*CCHa1R*. Aforementioned protocols were followed to isolate total RNA and generate first-strand cDNA. qRT-PCR was conducted using an Mx3000P Light Cycler^®^ 96 system from Roche (Indianapolis, IN, USA). We prepared the reaction mixture using 5 μL of TB GreenTM Premix Ex TaqTM 11 (Takara), 1 μL of cDNA (≈200 ng/μL), 1 μL of each primer (10 μM), and 2 μL of nuclease-free water. We used the following PCR protocol: 3 min at 95 °C for initial denaturation, then 35 cycles of 10 s at 95 °C and 30 s at 60 °C. Sample integrity was assessed through a melting curve analysis, with temperatures ranging from 55 to 95 °C. The primer sequences utilized for qRT-PCR analysis are documented in (Table S1). For the reference gene, we chose ribosomal protein RPL7 (NM_001135898.1) [31] and analyzed the relative quantification of expression by using the 2^−∆Ct^ procedure. To verify primer specificity, we conducted melting curve analyses, seeking solitary peaks that signified exclusive amplification of target genes. The equation E = 10 − 1/slope was employed to calculate the amplification efficiency (E-value) from the linear standard curve. The efficiency of the reaction was found to be superior to 90%, validating our experimental setup. The qRT-PCR analysis was conducted using three biological replicates to ensure the reliability and robustness of the obtained results.

### 2.6. CCHa1/CCHa1R Expression Profiles Across Developmental Stages and Different Tissues

We assessed *CCHa1*/*CCHa1R* transcription across different developmental stages of *A. pisum* by sampling 20–30 individuals from each nymphal instar (first–fourth) and the adult stage. We performed this with three biological replicates for each stage. The aforementioned protocols were followed to isolate total RNA and generate first-strand cDNA. We dissected tissues from synchronized wingless adult aphids to measure *CCHa1*/*CCHa1R* transcription levels. Tissue samples included antennae (200 aphids), gut (100 aphids), brain (90–100 aphids), cuticle (15–20 aphids), and embryos (10 aphids), and all samples were prepared in 0.1 M PBS (pH 7.4) under a stereoscopic microscope. Following the previously described protocols, we performed total RNA extraction and first-strand cDNA synthesis. The experimental protocol maintained consistency by using three independent biological replicates throughout all procedures and analyses.

### 2.7. Profiling CCHa1/CCHa1R Expression in Response to Feeding and Starvation

The transcriptional profiles of the neuropeptide *CCHa1*/*CCHa1R* in adults (full body) and in the brains of *A. pisum* were measured following starvation and feeding treatment for 3 and 6 h. We adapted the previously described feeding protocol with minor adjustments for our experimental needs [17,32]. In brief, aphids assigned to the “starved” group received no food, while those in the “fed” group were provided with food. For treatment durations of 3 and 6 h, we employed fifteen adult aphids, utilizing five aphids for each individual replication under both the starvation and feeding conditions. Likewise, when focusing on the brain tissues, we dissected 50 brains for each biological replication after 3 and 6 h of starvation and feeding. In total, three biological replicates were prepared for both the 3 and 6 h treatments of starved and fed aphids. The dissections and subsequent experiments were carried out in a controlled environment using 0.1 M PBS (pH 7.4) under a stereoscopic microscope (Olympus). The processes for total RNA extraction and first-strand cDNA synthesis were conducted as mentioned above. The experimental protocol maintained consistency by using three independent biological replicates throughout all procedures and analysis.

### 2.8. Synthesis and Injection of dsRNA

Double-stranded RNA for *CCHa1* (259 bp) and *CCHa1R* (384 bp) was produced using adult aphid cDNA and the Transcript Aid T7 High Yield Transcription Kit (Thermo Fisher Scientific), adhering to the supplier’s instructions. We appended the T7 RNA polymerase promoter sequence (5′-TAATACGACTCACTATAGGG-3′) to both forward and reverse primers to enable dsRNA synthesis, as listed in Table S1. The PCR process involved an initial step at 95 °C for 3 min, followed by 35 cycles at 95 °C for 30 s, 60 °C for 30 s, and 72 °C for 45 s, concluding with a final polymerization step at 72 °C for 10 min. Following amplification, the amplicons were purified using the SteadyPure PCR DNA Purification Kit (Accurate Biotechnology in Changsha, Hunan, China). For dsRNA purification, we employed a phenol (pH 4.7) chloroform extraction and ethanol precipitation method, subsequently eluting the purified dsRNA in diethylpyrocarbonate (DEPC)-treated nuclease-free water. We synthesized a 714 bp *GFP* dsRNA fragment as a control, employing the aforementioned conditions and primers listed in Table S1. To assess dsRNA quantity, we employed a NanoDrop 2000 spectrophotometer from Thermo Fisher, while the integrity of the dsRNA was verified through gel electrophoresis using a 1% agarose gel prior to injection. A clear and singular band observed during gel electrophoresis confirmed the high quality of the dsRNA, indicating its suitability for injection into *A. pisum* for RNAi. We diluted the dsRNA to the desired concentration and preserved it at −80 °C until needed.

We administered dsRNA using an M3301 Nanoliter20 microinjector (World Precision Instruments, Sarasota, FL, USA). To facilitate the immobilization of aphids, a 1% agarose solution was prepared, allowed to cool until solid, and then formed into a petri dish. To secure the aphids, we etched two intersecting grooves into the agarose. Concurrently, we fabricated glass capillaries using 3.5-inch 3-000-203-G/X micropipettes (Drummond Scientific) and a PC-100 Dual-Stage Glass Micropipette Puller (Narishige). Prior to the micro-injection, aphids were immobilized by a brief 5 min incubation on ice. Subsequently, they were positioned on the agarose plate, and approximately 300 nL of dsRNA (at a concentration of approximately 2.8 µg/µL), targeting *dsCCHa1*, *dsCCHa1R*, and *dsGFP* as a control. The injection was delivered to the lateral thorax of adult aphids, specifically between the middle and posterior leg pairs, following the methodology described by [33]. Adult aphids, aged 24 h, were selected for dsRNA injection. To visualize the distribution of injected material, we combined food dye with *dsGFP*, *dsCCHa1*, and *dsCCHa1R* before administering them to the aphids. Each treatment group consisted of 25 injected aphids. Following injection, the aphids were transferred to clip-caged *V. faba* leaves. Aphids were collected after 24 and 48 h.

### 2.9. Feeding Behavior Assay

We employed the EPG technique to analyze *A. pisum* feeding behavior across three groups: *dsCCHa1*, *dsCCHa1R* treatments, and *dsGFP* as a control [34], following the recently used protocol for *A. pisum* [35]. Fourteen hours post-injection, we attached a gold wire electrode (2 cm × 18 µm) to the dorsum of eight randomly selected aphids, securing it with electrically conductive silver glue. A Giga-8 DC EPG system served as the recording platform for the connected electrodes [34]. Stylet+, an integrated hardware–software solution from EPG Systems (Wageningen, The Netherlands), was employed to record EPG output. We established the plant connectivity by inserting an electrode into the potted plant’s soil, then shielded the entire experimental apparatus within a Faraday cage to mitigate electromagnetic interference. We conducted 8 h EPG recordings of aphids placed on the abaxial surface of a mature leaf from a 2-week-old faba bean plant. Each plant was used once in each replication, ensuring data accuracy and reducing potential biases. EPG waveforms were analyzed using the Stylet+ analysis module [36]. Waveforms were divided into two phases: the first phase was from the initiation of EPG to reaching the phloem, and the second phase related to phloem activity. In phloem-related activity, two waves, E1 and E2, mainly represent the feeding duration. The E2 wave represents the feeding duration and the E1 wave represents the pre-feeding duration. Further analysis was performed using the Excel Workbook for automatic parameter calculation of EPG data 4.3 [37]. Twenty replications were conducted to compare the feeding behavior of treated and control *A. pisum*.

### 2.10. Survival and Reproduction Assay

Following the injection of *dsCCHa1*, *dsCCHa1R*, and *dsGFP*, we performed a reproductive performance assessment. Non-injected aphids used a control. After dsRNA injection, the aphids were reared on fresh leaves inside the clip cage, and we recorded the fecundity and mortality of adults continuously after a 12 h duration from the beginning of the RNAi assay. To assess viability, 20 aphids per treatment group were subjected to a parallel survival assay. Following dsRNA injection, aphids were transferred to clip-caged *V. faba* leaves, where adult mortality was recorded daily.

### 2.11. Statistical Analysis

Different developmental stages and body tissue data were analyzed by one-way ANOVA, and post hoc comparisons of the means were performed using the Tukey test at the *p* < 0.05 level. Starvation, feeding, gene silencing, and EPG recording data were analyzed using Excel 2016 and SPSS 20 for comprehensive data evaluation. Statistical comparisons between the treatment and control groups were conducted using independent Student’s *t*-tests. Kaplan–Meier survival log rank analysis was applied to mortality data, and reproduction results were examined via independent Student’s *t*-tests.

## 3. Results

### 3.1. Characterization of CCHa1/CCHa1R and Molecular Docking Analysis

**Figure 1:**
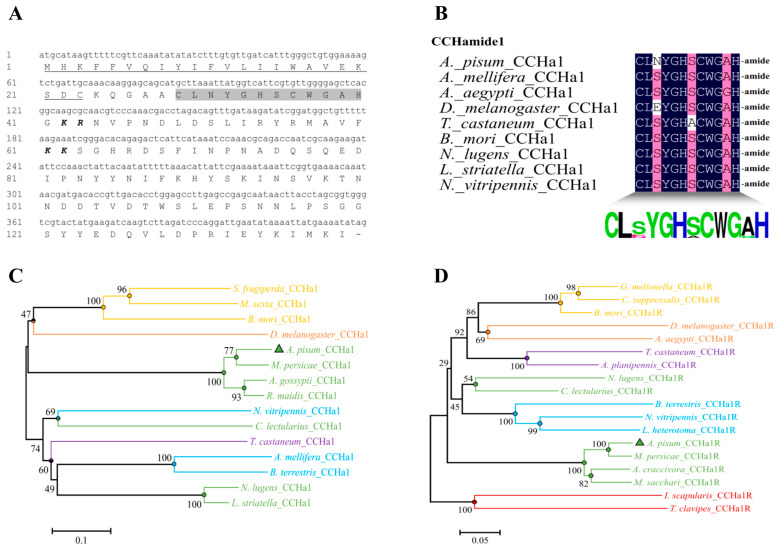
Bioinformatics analysis of *CCHa1*/*CCHa1R* from *A. pisum*. (**A**) Nucleotide and amino acid sequences of *CCHa1* cDNA. The underlined letters are predicted signal peptides, grey background amino acids represent mature peptides, and italic and bold letters are the dibasic cleavage sites. (**B**) Amino acid sequence alignment of *CCHa1* with other insect species; at the bottom, there is a consensus logo. (**C**,**D**) Phylogenetic tree of *CCHa1*/*CCHa1R* in different insects was constructed in MEGA 5.2 with 1000 bootstrap replicates. The model chooses P-distance and the Gaps/Missing DATA Treatment chooses Pairwise deletion. Numbers at branches are percent bootstrap support values. Accession numbers of *CCHa1*/*CCHa1R* are mentioned in Tables S2 and S3.

The amino acid and nucleotide sequences of the *CCHa1* ORF are depicted in (Figure 1A). The multi-species alignment of *CCHa1* peptides is presented in (Figure 1B), highlighting a shared [CLSYGHSCWGAH-amide] consensus motif at the C-terminus of the *CCHa1* neuropeptide family. The *A. pisum* genome database (https://bipaa.genouest.org/is/aphidbase/ accessed on 11 January 2024) was used to identify transcripts of the *CCHa1*-R, which has the GenBank accession number ACYPI071161. Characterized as a typical GPCR, the *A. pisum CCHa1R* features seven TM and encodes a protein consisting of 413 amino acids. Analysis of evolutionary relationships demonstrated that *A. pisum CCHa1*/*CCHa1R* are phylogenetically close to their homologs in various insect species. (Figure 1C,D).

**Figure 2:**
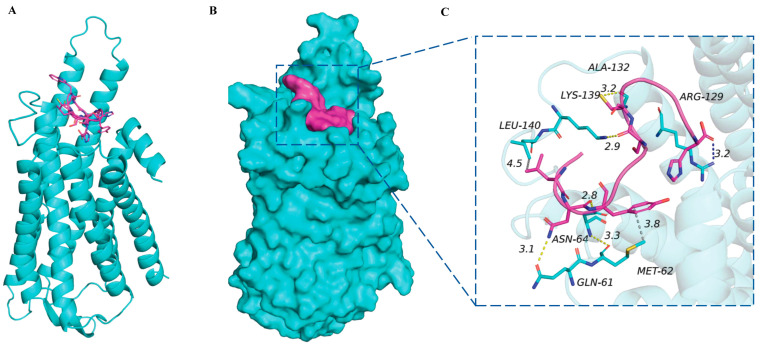
Docking analysis of *CCHa1* mature peptide and its receptor *CCHa1R*. (**A**) Ribbon diagram represents the *CCHa1R* protein bound to the *CCHa1* peptide (shown in purple), illustrating the overall docking pose and spatial orientation within the receptor’s binding pocket. (**B**) Surface representation of the *CCHa1R* protein with the *CCHa1* peptide (highlighted in purple) positioned within the binding pocket. The interaction interface between the receptor and peptide. (**C**) Close-up view of the binding interactions between the *CCHa1* peptide and key residues within the receptor’s binding site. Hydrogen bonds and distances between critical amino acids (e.g., ARG-129, ALA-132, LYS-139) are indicated, highlighting the molecular contacts involved in the docking. Yellow dash represents hydrogen bond distance.

An alignment was conducted between *A. pisum CCHa1R* and the mRNA sequences of nine other *CCHa1R* insects. *A. pisum CCHa1R* showed identity with other receptors, such as the four *CCHa1R* hemipterans (*Aphis craccivora* 69.84%; *Diaphorina citri* 42.41%; *Bemisia tabaci* 34.05%; *Nilaparvata lugens* 41.25%), three coleopterans (*Tribolium castaneum* 37.94%; *Agrilus planipennis* 37.16%; *Tilloclytus clavipes* 29.77%), two dipterans (*D. melanogaster* 39.11%; *Aedes aegypti* 38.91%), one hymenopteran (*Nasonia vitripennis* 40.08%), one lepidopteran (*B. mori* 38.91%), and one ixodidae (*Ixodes scapularis* 31.13%). The most conserved portions of these GPCRs were predominantly found within their transmembrane segments (Figure S1). The *A. pisum CCHa1* mature peptide (CLNYGHSCWGAH) was docked to the *CCHa1R* target protein. Our molecular docking simulations revealed strong peptide–protein interactions, evidenced by a high binding energy of −8.149 kcal/mol. The *CCHa1R* protein binds the *CCHa1* mature peptide using the following residues: ARG-129, LYS-139, GLN-61, ASN-64, ALA-132, LEU-140, and MET-62. For the *CCHa1* mature peptide, the following residues are involved in the binding: HIS-12, SER-7, ASN-3, LEU-2, CYS-8, and TYR-4 (Figure 2).

### 3.2. Transcriptional Expression of CCHa1/CCHa1R During Developmental Stages and in the Body Tissues of A. pisum

**Figure 3:**
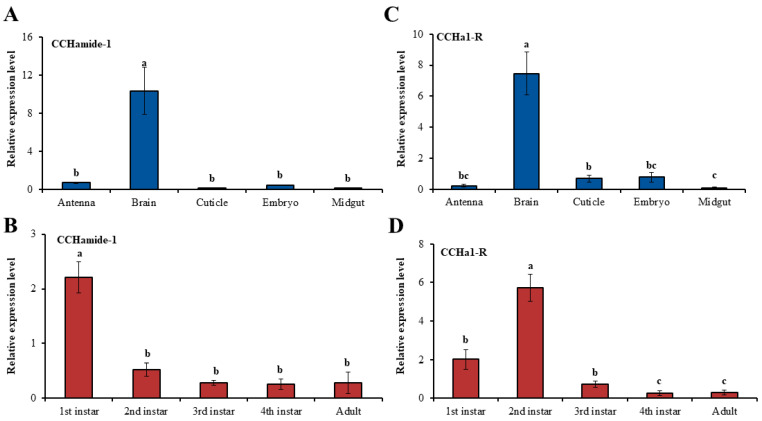
Relative expression levels of *CCHa1*/*CCHa1R* in different body parts during different developmental stages. (**A**–**C**) Relative expression levels of *CCHa1*/*CCHa1R* transcript in different body parts. (**B**–**D**) Relative expression levels of *CCHa1*/*CCHa1R* transcript at different developmental stages. (**A**–**D**) For each sample, the transcript level was measured via qRT-PCR and normalized against RPL7. The bars correspond to the average of three independent replicates. Results are shown as means ± S.E. The data were statistically analyzed by one-way ANOVA followed by Tukey test. Different lowercase letters indicate significant differences at the 0.05 level.

Transcript abundance of *CCHa1*/*CCHa1R* was quantified across various developmental stages and body tissues using qRT-PCR. First-instar nymphs exhibited the highest expression level, while *CCHa1R* was detected as high in the 2nd-instar nymphs (Figure 3B,D). *A. pisum*’s antennae, brains, cuticles, embryos, and midguts exhibited diverse expression levels for *CCHa1*/*CCHa1R*, indicating tissue-specific regulation (Figure 3A,C). The brain exhibited the highest expression of both *CCHa1*/*CCHa1R*, while *CCHa1* was notably absent in the antennae, cuticles, and guts (Ct values ≥ 29). Low expression levels were recorded in embryo samples, possibly due to the diluting effect of the included head tissue.

### 3.3. Transcriptional Expressional Levels of CCHa1/CCHa1R Under Starvation and Feeding Conditions of A. pisum

**Figure 4:**
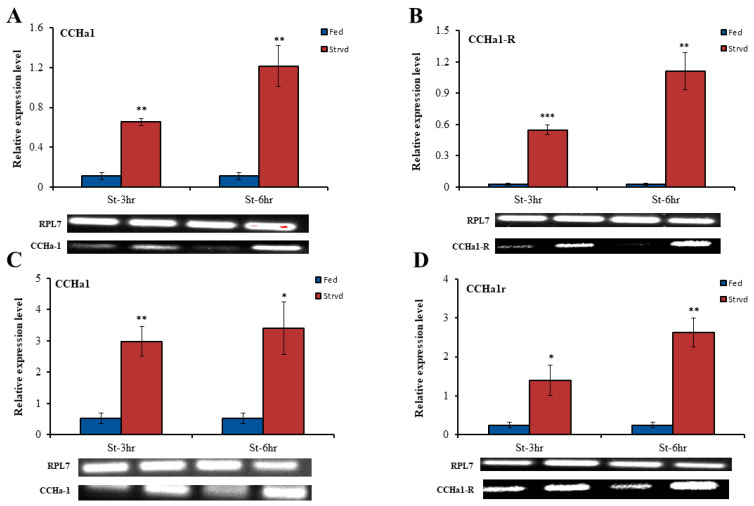
Effect of starvation on the transcriptional expression of *CCHa1*/*CCHa1R* in adults and brain samples. (**A**,**B**) Transcriptional expression of *CCHa1*/*CCHa1R* after starvation in adults. (**C**,**D**) Transcriptional expression of *CCHa1*/*CCHa1R* after starvation in brain. (**A**–**D**) Data were generated from three independent biological replications. Results are shown as means ± S.E. The asterisk on each bar represents the significant difference compared to fed aphids, calculated by statistical analysis (independent student t test, * *p* < 0.05, ** *p* < 0.01, *** *p* < 0.001, respectively).

We measured the transcript expression of *CCHa1*/*CCHa1R* in both fed and starved aphids, revealing significant disparities between the two groups. Remarkably, our findings showed a substantial upregulation of *CCHa1*/*CCHa1R* gene expression in starved aphids as compared to their fed counterparts (Figure 4A,B).

Moreover, the transcripts for *CCHa1*/*CCHa1R* were primarily localized in the brain (Figure 3A,C); our investigation also focused on the relative expression within the brains of fed and starved aphids. Intriguingly, our observations revealed a similar upregulated expression pattern for *CCHa1*/*CCHa1R* in starved aphids as compared to the control groups (Figure 4C,D). Furthermore, the transcript expression levels of both *CCHa1*/*CCHa1R* displayed increases in response to prolonged starvation stress.

### 3.4. RNAi-Based Silencing of CCHa1/CCHa1R via dsRNA Injection

**Figure 5:**
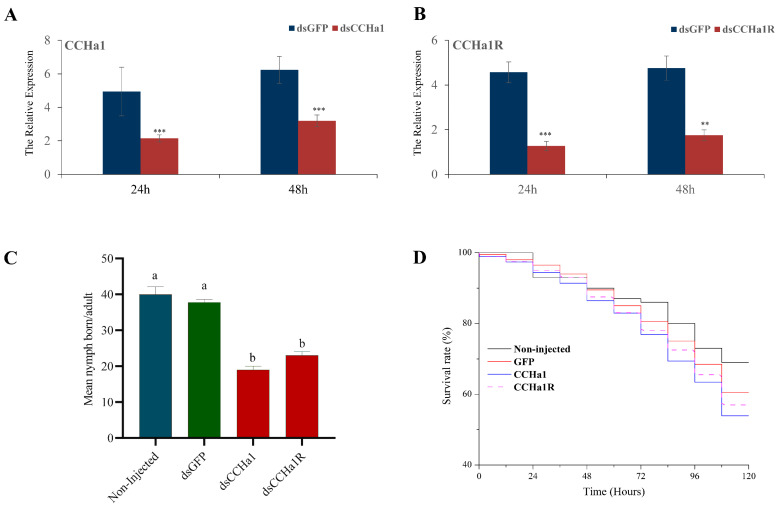
RNAi mediated knockdown of *CCHa1*/*CCHa1R* expression levels in *A. pisum*. (**A**,**B**) Gene silencing efficiency was detected at different sampling time-points (24 and 48 h) after injection of *dsCCHa1*, *dsCCHa1R* as treatment, and *dsGFP* as a control. (**C**) Effect of *CCHa1*/*CCHa1R* gene silencing on reproduction of *A. pisum* after injection of ds*CCHa1*, ds*CCHa1R*, and *dsGFP*, as well as in non-injected controls, respectively. (**D**) Influence of *dsCCHa1* and *dsCCHa1R* gene silencing on *A. pisum* survival. Kaplan–Meier survival log-rank analysis shows no significant difference in survival between the ds*CCHa1* and ds*CCHa1R* treatments and the *dsGFP*-injected control (*p* < 0.05). (**A**–**C**) Each treatment contained three biological replicates analyzed by qRT-PCR. The asterisk on each bar represents a significant difference compared to injected *dsGFP*, calculated by statistical analysis (independent Student’s *t* test, ** *p* < 0.01, *** *p* < 0.001, respectively).

One-day-old adult aphids were subjected to dsRNA injection. At the point of injection, living aphids exhibited cuticular melanization. Significant silencing of *CCHa1*/*CCHa1R* after dsRNA injection was achieved by 300 nL (∼3 µg/µL) of *dsCCHa1* and *dsCCHa1R* compared to *dsGFP* as a control. At 24 and 48 h after dsRNA injection, the expression of *dsCCHa1* and *dsCCHa1R* was downregulated (Figure 5A,B).

### 3.5. CCHa1/CCHa1R Knockdown Effects on A. pisum Feeding Behavior

EPG analysis was employed to examine changes in probing and feeding durations following RNAi-mediated silencing of *dsCCHa1* and *dsCCHa1R*, with *dsGFP* serving as a control. Our analysis focused on 27 EPG parameters: 11 for probing activities and 16 for plant phloem-related behaviors (Table 1). Regarding stylet activity before phloem access, measured by the first probe duration, it showed a marked reduction from 7 h (*dsGFP* control) to 5.6 h (*dsCCHa1*) and 6.1 h (*dsCCHa1R*). (*p* = 0.021). Similarly, both the number of probes and the overall duration of probing exhibited significant decreases across the two groups (*p* = 0.012 and *p* = 0.027). Simultaneously, the total and mean durations of C and np waves remained statistically similar across the groups (*p* = 0.066, 0.305 and *p* = 0.22, 0.490, respectively).

As aphids are plant phloem-sucking insects, the initial insertion of the stylet in the phloem was delayed from ~1.9 h (*dsGFP* group) to 3.4 h and 3.1 h in the *dsCCHa1* and *dsCCHa1R* groups, respectively, as mentioned by “Time from start of EPG to 1st E” (*p* = 0.046). While E and E2 waveforms showed reduced total durations (*p* = 0.001 and *p* = 0.014), respectively, the non-phloematic phase exhibited a significant prolonged time (*p* = 0.036). However, a significant decrease was observed in the frequency of both E1 and E2 waveforms (*p* = 0.015 and *p* = 0.003, respectively), and in the mean duration of the E1 or total duration of E1, no significant differences were observed (*p* = 0.682 and *p* = 0.560, respectively). Compared to the *dsGFP* control, both *dsCCHa1* and *dsCCHa1R* treatments showed significant decreases in multiple E2 waveform metrics: mean duration, longest duration, frequency of sustained events, and percentage of probing time (*p* = 0.001, *p* = 0.001, *p* = 0.016, and *p* = 0.010, respectively).

## 4. Discussion

In this study, we identified the sequences of *CCHa1*/*CCHa1R* in the pea aphid, *A. pisum*, revealing significant similarities with the corresponding precursors and receptors from other insect species. Notably, the transmembrane domain regions of *CCHa1R* exhibited significant conservation across different species. Phylogenetic analysis of *CCHa1R* revealed a higher degree of similarity with receptors from other insects compared to the sequence identity observed in the alignment. The reason for this difference is that identity quantifies the exact matches of residues—either nucleotide or amino acid—between two sequences, while similarity indicates the extent of resemblance based on functional, structural, or evolutionary connections [17]. The docking analysis revealed that the *CCHa1* peptide binds to its receptor *CCHa1R* through specific amino acid residues, which are critical for maintaining the structural and functional integrity of the peptide–receptor complex. Residues such as HIS-12, SER-7, ASN-3, LEU-2, CYS-8, and TYR-4 in the peptide, as well as ARG-129, LYS-139, GLN-61, ASN-64, ALA-132, LEU-140, and MET-62 in the receptor, show strong interaction patterns, suggesting a high degree of specificity and affinity in the binding process (Figure 2). Moreover, in the peach–potato aphid, the binding of *AKH* and *AKHR* has been demonstrated and the binding between the receptor and 2271 analog has been predicted to be more stable than the binding with the natural *AKH* [38].

The relative expression levels of *CCHa1*/*CCHa1R* transcript varied across different developmental stages in *A. pisum*, with a significantly higher expression observed in the first and second nymphal instars compared to the other stages (Figure 3B,D). It is important to note that the low expression levels of the receptor in the later nymphal instars make it difficult to detect any changes in expression through qRT-PCR, which may explain the lack of significant variations across different developmental stages. It was remarkable that neuropeptide receptors other than *CCHa1R* that are involved in feeding regulation in *A. pisum*, such as *CCHa2-R*, neuropeptide F receptor (NPFR), and short neuropeptide F receptor (sNPFR), also displayed consistent expression levels across different developmental stages [17,32,35].

*CCHa1*/*CCHa1R* mRNA levels were significantly upregulated in the brains of *A. pisum*, implying that cells located in the guts, antennae, and cuticles of the aphids are not the primary sites of *CCHa1*/*CCHa1R*. Our findings were similar with previously reported data on receptors in *A. pisum*, showing that *CCHa2-R* expression is more pronounced in the brain compared to the gut and other body tissues [17]. Our findings corroborate the preexisting observations from other insect species and show that in *D. melanogaster*, both *CCHa1* and *CCHa2* are primarily synthesized and secreted in the brain and midgut endocrine cells [15,18,39], and so in turn, these peptides are named “brain-gut peptides” (enteroendocrine peptides). *A. aegypti* transcript expression of *CCHa1* was found to be higher in the head, midgut, and carcass (cuticle, fat body, and skeletal muscle) [23]. Particularly, when assessing sex differences, the transcript of *CCHa1* exhibited increased levels in the midgut of male mosquitoes as compared to the head [23]. However, comparable expression of *CCHa1* was observed in the female head and midgut [23]. Moreover, the immunomapping of *CCHa1* in *A. aegypti* detected neurons in the brain, the ventral nerve cord, and enteroendocrine cells in the posterior midgut close to the midgut/hindgut junction, confirming the expression profiles of these transcripts [23]. Considering the differences in *CCHa1* transcript levels between the heads and midguts of male and female *A. aegypti*, we speculate that the exclusive presence of *CCHa1*/*CCHa1R* only in the brain may be related to the parthenogenetic characteristics of *A. pisum*.

Starvation influences the expression levels of *CCHa1*/*CCHa1R* in adults and in the brains of *A. pisum* (Figure 4A–D). The *CCHa1* signaling cascade has documented functions closely related to the feeding and nutritional statuses of diverse insect species. Therefore, we set specific time points to monitor changes in the expression levels of *CCHa1*/*CCHa1R* in *A. pisum* under conditions of starvation stress and feeding. Simultaneous collection of aphids across all experimental groups was performed to prevent the circadian rhythm from influencing *CCHa1R* transcript expression levels. In *A. pisum*, starvation induced an upregulation of *CCHa1*/*CCHa1R* transcript levels, whereas feeding triggered a downregulation, irrespective of quantitative differences between time points. Our findings demonstrated that the expression of *CCHa1*/*CCHa1R* transcripts in *A. pisum* is influenced by the organism’s feeding state. Moreover, our findings were consistent with other feeding-modulated neuropeptides in hemipterans, including *sNPF* and *NPF*, where the transcript expression of precursors and receptors is downregulated under starvation stress and returns to baseline following refeeding [40]. However, in chewing mouthparts insects, including *Schistocerca gregaria* and *B. mori*, starvation-induced stress downregulates the transcript expression of *sNPF* (documented role in feeding in both species) [41]. The correlation between starvation stress and transcript expression may stem from the species-specific physiology of feeding behavior [35]. Taken together, our observations suggest that the *CCHa1* signaling pathway is implicated in the nutritional response of wingless parthenogenetic aphids, particularly through the regulation of *CCHa1R* expression.

The putative functions of *CCHa1*/*CCHa1R* in regulating *A. pisum* feeding were analyzed using RNAi. Effective target gene silencing requires the incorporation of dsRNA molecules into the insect body, leading to reduced gene expression at the transcription stage [42]. In *A. pisum*, the brain exhibits a higher expression level of *CCHa1*/*CCHa1R* than other tissues (Figure 3A,C). Following RNAi injection, we observed up to a 50% reduction in *CCHa1*/*CCHa1R* mRNA levels across the whole aphid body (Figure 5A,B). The significant reduction in gene expression at 24 and 48 h after *CCHa1*/*CCHa1R* silencing prompted us to analyze alterations in *A. pisum* feeding behavior following dsRNA injection. Ultimately, we employed the EPG technology to investigate changes in *A. pisum* feeding behavior following *CCHa1*/*CCHa1R* knockdown. Our findings showed that suppressing *CCHa1*/*CCHa1R* expression in *A. pisum* inhibited the appetite and reduced the food intake of the aphids (Table 1). This suggests that silencing these genes regulates the aphid’s appetite, potentially involving *NPF*, *sNPF*/*sNPFR*, and *CCHa2R* pathways, which consequently modifies feeding behavior and effectively decreases food consumption [17,32,35]. The appearance and length of the E waveform in EPG studies generally reflected the aphid’s prioritization of phloem sap feeding [36]. Consequently, the observed delay in phloem activity among *A. pisum* treated with *dsCCHa1* and *dsCCHa1R* indicated a reduced appetite. Corroborating this, *dsCCHa1*-treated aphids also showed a reduced duration of initial probing and overall probing time. Primarily, the reduced appetite and shortened phloem ingestion duration contribute to the decrease in food intake observed in *dsCCHa1*-silenced aphids (Table 1). Our findings revealed a correlation between *CCHa1/CCHa1R* transcript abundance and feeding behavior in *A. pisum*; however, the precise downstream signaling mechanism is yet to be elucidated. Nonetheless, considering the high level of *CCHamide-1* and *CCHa1R* mRNA in the brain of *A. pisum*, we speculate that the brain synthesizes and releases a signal to the *CCHa1R* to stimulate food intake.

The suppression of *CCHa1*/*CCHa1R* expression has been observed to influence the reproductive process in aphids. The reproduction assay showed a significant decline in aphid fecundity following injection of *dsCCHa1* and *dsCCHa1R*, although survival was not notably influenced by the *CCHa1*/*CCHa1R* knockdown. The results suggested that aphids might compromise their reproductive capacity to maintain survival in response to nutrient scarcity [43]. Previous studies on *CCHa2R*, *NPF*, and *sNPF*/*sNPFR* also showed that the survival rate of *A. pisum* remained unaffected, showing no significant changes [17,32,35]. An additional factor potentially contributing to the significant decrease in reproduction after RNAi of *CCHa1*/*CCHa1R* might be a consequence of *CCHa1*’s indirect involvement in the lipid metabolic process. Indeed, reproductive success is closely tied to lipid availability, as these compounds serve as the main energy source, with reduced levels leading to diminished reproductive output [44]. As this effect has not been previously reported in *A. pisum*, further investigation into the impact of *CCHa1* on lipid metabolism is needed.

In conclusion, in *A. pisum*, the *CCHa1*/*CCHa1R* transcripts were found to be highly expressed in the brain. Our findings suggest that *CCHa1*/*CCHa1R* play a role in modulating aphid feeding patterns and food consumption, with subsequent effects on reproduction, but not on survival. Furthermore, the addition of *CCHa1*/*CCHa1R* to the existing *A. pisum* “feeding toolkit” represents a valuable contribution to our understanding of insect feeding behavior, particularly in *A. pisum*. While our study centered on wingless *A. pisum*, it is important to note that aphids exhibit wing dimorphism—a trait that allows them to adapt to environmental changes. Winged and wingless aphids display distinct morphological, physiological, and behavioral characteristics [45]. Nutrient availability is believed to be a pivotal factor in orchestrating the wingless-to-winged transition in aphids [46]. Therefore, we believe that investigation into the role of *CCHa1*/*CCHa1R* in winged *A. pisum* feeding behavior would provide valuable insights.

## Figures and Tables

**Table 1:** Comparison of probing and feeding behaviors of *Acyrthosiphon pisum* in the *dsCCHa1*, *dsCCHa1R* treatment, and *dsGFP* control groups on broad bean seedlings based on EPG recordings.

Tissue Specificity	Parameters		*dsGFP*	*dsCCHa1*	*dsCCHa1R*	*p*
		N	Mean [s] ± SE	Mean [s] ± SE	Mean [s] ± SE	
(From Initiation of EPG to Reaching Phloem)						
Epidermis	Duration of 1st probe	20	24915 ± 1294 a	20,443.21 ± 1105 b	21,985 ± 912 ab	0.021
Epidermis and mesophyll	Time from 1st probe to 1st E	20	6595 ± 1101 b	11,256 ± 1636 a	10,040 ± 1443 ab	0.029
All tissues	Time from start of EPG to 1st sustained E2 (>10 min)	20	8647 ± 1107 b	14,304.79 ± 2066 a	13,925.73 ± 1904 ab	0.044
All tissues	Number of probes	20	4.35 ± 0.76 a	2.3 ± 0.19 b	2.95 ± 0.26 b	0.012
All tissues	Total probing time	20	27,635 ± 447 a	25,839 ± 486 b	26,197 ± 523 b	0.027
All tissues	Number of C	20	9.75 ± 4.24 a	7.58 ± 2.21 b	7.65 ± 2.53 b	0.035
All tissues	Total duration of C	20	21,222 ± 1005 b	24,078 ± 713 ab	24,272 ± 1247 a	0.066
All tissues	Mean duration of C	20	4806 ± 501 a	3896 ± 337 a	3993 ± 340 a	0.22
All tissues	Number of np	20	2.15 ± 0.29 b	3.1 ± 0.31 a	2.95 ± 0.23 a	0.042
All tissues	Total duration of np	20	466 ± 90 a	666 ± 91 a	553 ± 92 a	0.305
All tissues	Mean duration of np	20	132 ± 26 a	176 ± 25 a	153 ± 27 a	0.490
(Related to Phloem Activity)						
Epidermis and mesophyll	Time from start of EPG to 1st E	20	7096 ± 994 b	12,379 ± 1866 a	11,248 ± 1640 ab	0.046
All tissues	Time from start of EPG to 1st E2	20	7600 ± 1091 b	13,544 ± 1322 a	11,472 ± 1397 ab	0.006
All tissues	Time from 1st probe to 1st sustained E2 (>10 min)	20	7862 ± 815 b	13,813 ± 1871 a	13,012 ± 2145 ab	0.034
Phloem	Total duration of E1 followed by E2 and E2	20	12,159 ± 770 a	8139 ± 1025 b	8671 ± 1011 b	0.007
Phloem	Number of single E1	20	0.6 ± 0.2 b	2.55 ± 0.7 a	2.15 ± 0.5 ab	0.043
Phloem	Number of E1	20	7.5 ± 0.7 a	5.35 ± 0.5 b	5.5 ± 0.4 b	0.015
Phloem	Number of E2	20	6.15 ± 0.5 a	4.1 ± 0.2 b	4.65 ± 0.3 b	0.003
Phloem	Number of sustained E2 (longer than 10 min)	20	4.15 ± 0.3 a	2.8 ± 0.3 b	3.01 ± 0.3 b	0.016
Phloem	Total duration of E	20	11,598 ± 638 a	7036 ± 922 b	8340 ± 936 b	0.001
Phloem	Total duration of E1	20	495 ± 103 a	378 ± 62.1 a	414 ± 60.9 a	0.560
Phloem	Total duration of E2	20	10,320 ± 817 a	6707 ± 963 b	7175 ± 969 b	0.014
Phloem	Mean duration of E1	20	98.3 ± 9.2 a	88.0 ± 8.4 a	90.3 ± 8.3 a	0.682
Phloem	Mean duration of E2	20	2610 ± 330 a	1270 ± 182 b	1491 ± 252 b	0.001
Phloem	Duration of 1st E	20	3254 ± 430 a	1335 ± 291 b	1492 ± 299 b	0.001
Phloem	Duration of the longest E2	20	6763 ± 759 a	3867 ± 441 b	4136 ± 481 b	0.001
Phloem	% of probing spent in E2	20	31.73 ± 3.3 a	18.98 ± 3.3 b	21.26 ± 2.3 b	0.010

The two means with the same lowercase letters in the same row are not significantly different (Independent sample *t*-test *p* > 0.05).

## Data Availability

The data presented in this study are available upon request from the corresponding author.

## Supplementary Materials

The following supporting information can be downloaded at https://www.mdpi.com/article/10.3390/insects15120939/s1, Figure S1: Amino acid sequence alignment of the *A. pisum CCHa1R* with other related GPCRs. Seven transmembrane domains are highlighted with black lines (TM1–TM7). Table S1: PCR primers for qRT-PCR and dsRNA synthesis. Table S2: *CCHa1* Accession number of genes used for phylogenetic analysis. Table S3: *CCHa1R* Accession number of genes used for phylogenetic analysis.
