# Supplementary material for: RNAi of Neuropeptide CCHamide-1 and Its Receptor Indicates Role in Feeding Behavior in the Pea Aphid, Acyrthosiphon pisum
Source: Insects. 2024 Nov 28;15(12):939. doi: 10.3390/insects15120939 (PMC11678771; doi:10.3390/insects15120939)
Supplement: Supplementary file 1 [file insects-15-00939-s001.zip › insects-3338682-supplementary.pdf]

Supplementary data

RNAi of Neuropeptide CCHamide-1 and its Receptor Indicates Role in Feeding Behavior in the Pea Aphid, *Acyrthosiphon pisum*

Sohaib Shahid [1,2,3], Muhammad Bilal Amir [1], Tian-Bo Ding [1], Tong-Xian Liu [1,4], Guy Smagghe [4,5,6] and Yan Shi [1,*]

[1] Shandong Engineering Research Center for Environment-Friendly Agricultural Pest Management, College of Plant Health and Medicine, Qingdao Agricultural University, Qingdao 266109, P.R. China; sohaibshahid90@hotmail.com (S.S.); dr.mbilalamir@outlook.com (M.B.A.); tbding@qau.edu.cn (T.-B.D.); txliu@qau.edu.cn (T.-X.L.)

[2] State Key Laboratory of Integrated Management of Pest Insects and Rodents, Institute of Zoology, Chinese Academy of Sciences, Beijing 100101, P. R. China

[3] University of Chinese Academy of Sciences, Beijing 100049, P. R. China

[4] Institute of Entomology, Guizhou University, Guiyang 550025, China; guysma9@gmail.com

[5] Cellular and Molecular Life Sciences, Department of Biology, Vrije Universiteit Brussel (VUB), 1050 Brussels, Belgium

[6] Department of Plants and Crops, Ghent University, 9000 Ghent, Belgium

* Correspondence: shiyanyuanyi@aliyun.com

This file include:

Figure S1

Table S1-S3

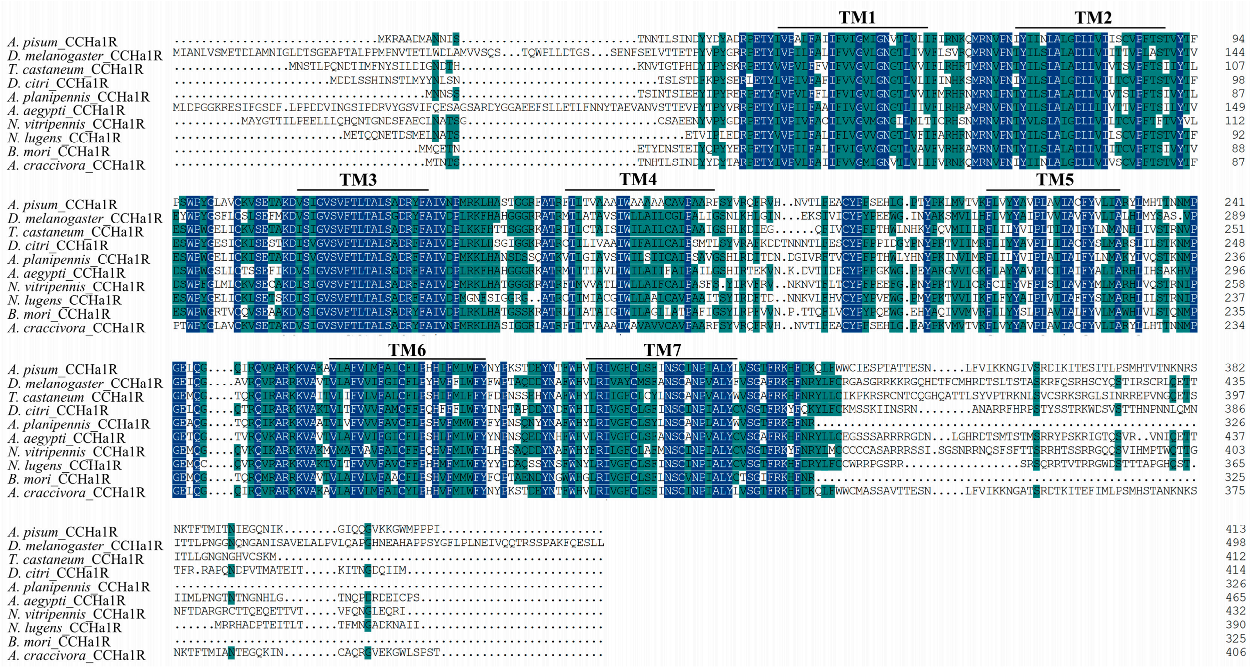

Figure S1 Amino acid sequence alignment of the *A. pisum* CCHa1-R with other related GPCRs. Seven transmembrane domains are highlighted with black line (**TM1-TM7**).

Table S1: PCR primers for qRT-PCR and dsRNA synthesis

Usage	Primer name	Forward primer (5-3)	Reverse primer (3-5)
qRT-PCR	CCHa-1	TGGGCTGTGGAAAAGTCTGA	CGATTGGTCTGCGTTTGGAT
qRT-PCR	CCHa1-R	TTTCCGTGTTCACGTTGACC	GGTAACATGCCTCGAACAGC
qRT-PCR	RPL7	GCGCGCCGAGGCTTAT	CCGGATTTCTTTGCATTTCTTG
dsRna synthesis	CCHa1	TAATACGACTCACTATAGGCCCAAACGACCTAGACAG	TAATACGACTCACTATAGGCCTGGGATCTAAGACTTGA
dsRna synthesis	CCHa1-R	TAATACGACTCACTATAGGGCCGACATGGCAAACAACAT	TAATACGACTCACTATAGGTGGCAAAATAACGGTCAGCG
dsRna synthesis	GFP	TAATACGACTCACTATAGGAAGGGCGAGGAGCTGTTCACCG	TAATACGACTCACTATAGGCAGCAGGACCATGTGATCGCGC

Table S2: CCHa1 Accession number of genes used for phylogenetic analysis

Species	Accession Number
Acyrthosiphon pisum	ACYPI071161
Apis mellifera	XP_625263
Bombus terrestris	XM_003397012.3
Nilaparvata lugens	AB817245.1
Laodelphax striatella	MF765468.1
Triboilum castaneum	NM_001293613.1
Nasonia vitripennis	XM_003425575.3
Cimex lectularius	XM_014406949.2
Drosophila melanogaster	NM_001104314.2
Bombyx mori	BMgn014466
Spodoptera frugiperda	XM_035585133.2
Manduca sexta	XM_030175029.2
Myzus persicae	XM_022317466.1
Aphis gossypii	XM_027985100.2
Rhopalosiphum maidis	XM_026951094.1

Table S3: CCHa1-R Accession number of genes used for phylogenetic analysis

Species	Accession Number
Acyrthosiphon pisum	XM_001947120.3
Nasonia vitripennis	XP_016845008.1
Leptopilina heterotoma	XM_043626751.1
Bombus terrestris	XM_048411468.1
Nilaparvata lugens	XM_022344998.1
Cimex lectularius	XM_024225334.1
Myzus persicae	XM_022318662.1
Aphis craccivora	KAF0773929.1
Melanaphis sacchari	XM_025334709.1
Tribolium castaneum	XM_008199257.2
Agrilus planipennis	A0A1W4X980
Drosophila melanogaster	A1ZAX0
Aedes aegypti	XP_021706929.1
Bombyx mori	BMgn009039
Galleria mellonella	XM_026900318.1
Chilo suppressalis	KT031013.1
Ixodes scapularis	XP_042147864.1
Trichonephila clavipes	PRD25357.1
